# The effect of the COVID-19 pandemic on the treated incidence of psychotic disorders in South London

**DOI:** 10.1192/j.eurpsy.2023.591

**Published:** 2023-07-19

**Authors:** D. Quattrone, E. Spinazzola, M. Di Forti, R. Murray

**Affiliations:** Institute of Psychiatry, King’s College London, London, United Kingdom

## Abstract

**Introduction:**

Evidence suggests that the COVID-19 pandemic was accompanied by an increased exposure to risk factors for psychosis, such as psychological stress, substance use, social isolation and deprivation, as well as a potential direct biological impact of the COVID-19 infection on psychosis. However, the impact of the COVID-19 pandemic on the rates of first-episode psychosis (FEP) has not yet been examined.

**Objectives:**

The aims of the current study were to 1) establish the FEP incidence between March 2019 and March 2021 in an inner London catchment area; 2) compare FEP incidence rates for the first year of the COVID-19 pandemic (March 2020 – February 2021), with those for the year prior (March 2019 – February 2020).

**Methods:**

We screened the clinical records of all individuals living in the London boroughs of Southwark and Lambeth who were referred to the early intervention in psychosis services before (from 1 March 2019 to 28 February 2020) and during (from 1 March 2020 to 28 February 2021) the COVID-19 pandemic. We used Office for National Statistics (ONS) data to estimate the risk populations stratified by sex and age group. We computed crude and sex-age standardised FEP incidence per 100,000 persons-year. We used Poisson regression to calculate the incidence rate ratio (IRR) before and during the COVID-19 pandemic and to examine the incidence variation by sociodemographic factors.

**Results:**

A total of 321 incident cases of FEP were identified during the COVID-19 pandemic accounting for a crude rate of 70.1 (95% CI 62.4 - 77.7) per 100,000 person-year. The crude rate for the year before was 47.5 (95% CI 41.2 - 53.8). The incidence variation between the two years accounted for an adjusted IRR of 1.46 (95% CI 1.23 – 1.76). The increased FEP rates were equally observed across the boroughs of Southwark and Lambeth and men and women. Individuals aged 20-24 (IRR 1.66; 95% CI 1.13 – 2.42) and those from the black ethnic group (IRR 1.61; 95% CI 1·24 – 2·09) showed the greatest incidence increases.

**Conclusions:**

To the best of our knowledge, this is the first study establishing the variation in FEP incidence before and during the COVID-19 pandemic across all adult age groups. We provide the first evidence that the COVID-19 pandemic resulted in a 46% increase in the incidence of psychotic disorders in South London. The increase was higher for young individuals and ethnic minorities. This finding should inform public health research and demonstrates the need for adequate resources for mental health secondary services.

**Disclosure of Interest:**

None Declared

